# Construction and validation of a prognosis signature based on the immune microenvironment in gastric cancer

**DOI:** 10.3389/fsurg.2023.1088292

**Published:** 2023-03-31

**Authors:** Li-Hong Wu, Xiang-Xu Wang, Yan Wang, Jing Wei, Zi-Rong Liang, Xi Yan, Jun Wang

**Affiliations:** ^1^ Xijing 986 Hospital Department, Fourth Military Medical University, Xi’an, China; ^2^ Xijing Hospital, Fourth Military Medical University, Xi’an, China

**Keywords:** immune microenvironment, gastric cancer, ssGSEA analysis, nomogram, prognosis signature

## Abstract

**Background:**

Gastric cancer (GC) is an aggressive malignant tumor with a high degree of heterogeneity, and its immune microenvironment is closely associated with tumor growth, development and drug resistance. Therefore, a classification system of gastric cancer based explicitly on the immune microenvironment context might enrich the strategy for gastric cancer prognosis and therapy.

**Methods:**

A total of 668 GC patients were collected from TCGA-STAD (*n* = 350), GSE15459 (*n* = 192), GSE57303 (*n* = 70) and GSE34942 (*n* = 56) datasets. Three immune-related subtypes (immunity-H, -M, and -L) were identified by hierarchical cluster analysis based on the ssGSEA score of 29 immune microenvironment-related gene sets. The immune microenvironment-related prognosis signature (IMPS) was constructed *via* univariate Cox regression, Lasso-Cox regression and multivariate Cox regression, and nomogram model combining IMPS and clinical variables was further constructed by the “rms” package. RT-PCR was applied to validate the expression of 7 IMPS genes between two human GC cell lines (AGS and MKN45) and one normal gastric epithelial cell line (GES-1).

**Results:**

The patients classified as immunity-H subtype exhibited highly expressed immune checkpoint and HLA-related genes, with enriched naïve B cells, M1 macrophages and CD8 T cells. We further constructed and validated a 7-gene (CTLA4, CLDN6, EMB, GPR15, ENTPD2, VWF and AKR1B1) prognosis signature, termed as IMPS. The patients with higher IMPS expression were more likely to be associated with higher pathology grade, more advanced TNM stages, higher T and N stage, and higher ratio of death. In addition, the prediction values of the combined nomogram in predicting 1-year (AUC = 0.750), 3-year (AUC = 0.764) and 5-year (AUC = 0.802) OS was higher than IMPS and individual clinical characteristics.

**Conclusions:**

The IMPS is a novel prognosis signature associated with the immune microenvironment and clinical characteristics. The IMPS and the combined nomogram model provide a relatively reliable predictive index for predicting the survival outcomes of gastric cancer.

## 1. Introduction

Gastric cancer (GC) is a disease with considerable heterogeneity (1), and is the fourth most fatal cancer worldwide (2). Patients with GC are rarely diagnosed at an early stage owing to insidious symptoms, and 25%–50% of patients with late-stage GC eventually emerge metastasis during the disease (3). The heterogeneity of the tumor microenvironment has been reported as a potential biomarker of various cancer prognoses (4, 5). It is worth noting that the tumor microenvironment (TME) components play a crucial role in tumor development. Therefore, the identification of tumor immunophenotypes might provide new insights for understanding tumor biology and cancer prognosis.

With the development of bioinformatics technology, the immune microenvironment components can be quantitatively analyzed (6). The single-sample gene set enrichment analysis (ssGSEA) (7) has been used to calculate quantitative scores for different types of immune genomics characteristics and to further classify the different immunophenotypes. In the present study, we identified three immunophenotypes (immunity-H, -M and -L) based on the characteristics of 29 immune-related gene sets. In addition, we constructed an immune microenvironment-related prognosis signature (IMPS) *via* univariate Cox regression, Lasso-Cox regression, and multivariate Cox regression, and validated the model in testing and independent cohorts. The patients with higher IMPS were associated with higher grade, advanced TNM stages, higher T and N stage, and death. We further constructed a nomogram model combining IMPS and clinical variables. The IMPS and the combined nomogram model have potential value in predicting survival outcomes of gastric cancer.

## 2. Materials and methods

### 2.1. Data obtaining and processing

A total of 668 GC patients were collected from TCGA-STAD (*n* = 350), GSE15459 (*n* = 192), GSE57303 (*n* = 70) and GSE34942 (*n* = 56) datasets. The transcriptome data (HTSeq-FPKM) and clinical information were downloaded from https://www.ncbi.nlm.nih.gov/geo/ and https://portal.gdc.cancer.gov/. The “limma” package was applied to perform deduplication and log2(x + 1) normalization processing on gene expression data (8).

### 2.2. Identification of three immune subtypes by hierarchical clustering analysis

The 29 immune microenvironment-related gene sets were collected from published literature (9). The ssGSEA algorithm *via* “GSVA” package was applied to evaluate the enrichment score of immunological characteristics in each GC sample (10).

### 2.3. Calculation of tumor purity and immune cell subpopulations

The package “ESTIMATE” was used to evaluate the stromal and immune cell components in the malignant tumor tissue to further estimate the total immune matrix scores, namely the Immune-, Stromal-, Estimate-scores, and to further evaluate the tumor purity (11). The subpopulations of 22 human immune cells were estimated by the relative subpopulations of RNA transcripts for cell type identification (CIBERORT) (12).

### 2.4. Screen of differentially expressed genes (DEGs) and function enrichment analysis

The DEGs between immunity-H and -L subtypes were screened *via* “limma” package with the screening conditions: |log FC| > 1, *P *< 0.05 (13). The heatmap and volcano map were depicted to present the differentially expressed genes, and the GO enrichment circle diagrams performed *via* the “GOplot” R package were used to present the functional enrichment analysis of DEGs (14).

### 2.5. Construction and validation of the immune microenvironment-related prognosis signature (IMPS)

The 350 GC samples in the TCGA-STAD cohort were randomly divided into TCGA-training (*n* = 246) and TCGA-testing cohorts (*n* = 104) *via* the “cart” package with a ratio of 7 : 3 (15). The GSE15459 (*n* = 193) and GSE57303 (*n* = 70) cohorts were used as independent validations. In the TCGA-training cohort, we first screened the prognostic-related genes by univariate Cox regression (*P* < 0.05), and further eliminate the collinearity among genes by Lasso-Cox regression analysis (16). Finally, we constructed a 7-gene prognosis signature *via* multivariate Cox analysis with the “stepwise regression method” (17). The IMPS of GC prognosis was calculated by the following formula: IMPS = −0.53 * CTLA4 + 0.12 * CLDN6 + 0.18 * EMB + 0.2 * GPR15 −0.14 * ENTPD2 + 0.22 * VWF + 0.25 * AKR1B1. The prognosis value of these 7 hub genes and the IMPS were further validated in both TCGA-testing and GSE15459 cohorts.

### 2.6. Prediction value of IMPS in immunotherapy response

Due to the lack of clinical studies on gastric cancer immunotherapy including mRNA sequencing data, we used the IMvigor210 cohort (18) (metastatic urothelial carcinoma) and Riaz-2017 cohort (19) (advanced melanoma) to verify the potential predictive value of IMPS in the outcome of immunotherapy response.

### 2.7. Quantitative real-time PCR

Two human GC cell lines (AGS and MKN45) and one normal gastric epithelial cell line (GES-1) were cultured for testing the expression of 7 IMPS genes. In addition, we further preformed the qPCR verification of seven Hub genes in five benign and five GC tissue samples. TRIzol (Servicebio, China) and PrimerScript™ RT Reagent kit (Takara, Tokyo, Japan) were used to extract total RNA and to further create the cDNA. The Real-Time PCR was performed by SYBR Green with the Real-Time PCR System (Roche, USA). The primers of the 7 hub genes were listed in Table 1. Finally, the RNA expression was normalized to GAPDH.

**Table 1:** Primer sequences of 7 hub genes.

Hub genes	Primer sequences
CTLA4(human)-F	GCCCTGCACTCTCCTGTTTTT
CTLA4(human)-R	GGTTGCCGCACAGACTTCA
ENTPD2(human)-F	AGACAAGGAGAACGACACAGG
ENTPD2(human)-R	AGGCATCCAACAAGACTCTGG
CLDN6(human)-F	TGTTCGGCTTGCTGGTCTAC
CLDN6(human)-R	CGGGGATTAGCGTCAGGAC
EMB (human)-F	CTGAGGGAGCAGTCTCCACG
EMB (human)-R	TGTAAAAGGCGAATCTGGGGC
GPR15(human)-F	TTACTATGCTACGAGCCCAAACT
GPR15(human)-R	CTCCCATGAGAACAAGGTTCC
VWF (human)-F	CCGATGCAGCCTTTTCGGA
VWF (human)-R	TCCCCAAGATACACGGAGAGG
AKR1B1(human)-F	CTGGTGGATGAAGGGCTGG
AKR1B1(human)-R	GGGTGGCACTCAATCTGGTT

### 2.8. Construction and evaluation of the combined nomogram considering IMPS and clinical characteristics

The combined nomogram prognostic model was constructed by the “rms” package based on clinical characteristics and the IMPS (20). The clinical characteristics included age, gender, pathology grade and TNM stage. The performance of the combined nomogram was evaluated by calibration plot using bootstrap method (21). And the time-ROC curve was performed to compare the prediction value among the combined nomogram model, IMPS, and clinical characteristics.

### 2.9. Statistical analyses

All statistical analyses were performed with the R software (version 4.1.2). The Mann–Whitney *U* test was used to compare the distribution difference of immune cells among different immune subtypes (22). The Chi-Squared test was used to evaluate the balance of clinical baseline between the training and testing cohorts (23). The Log-Rank test was performed to assess the differences in overall survival between different subtypes. The two-sided test with *P*-value <0.05 was considered statistically significant.

## 3. Results

### 3.1. Identification of three immune subtypes based on the ssGSEA score of 29 immune-related gene sets

**Figure 1:**
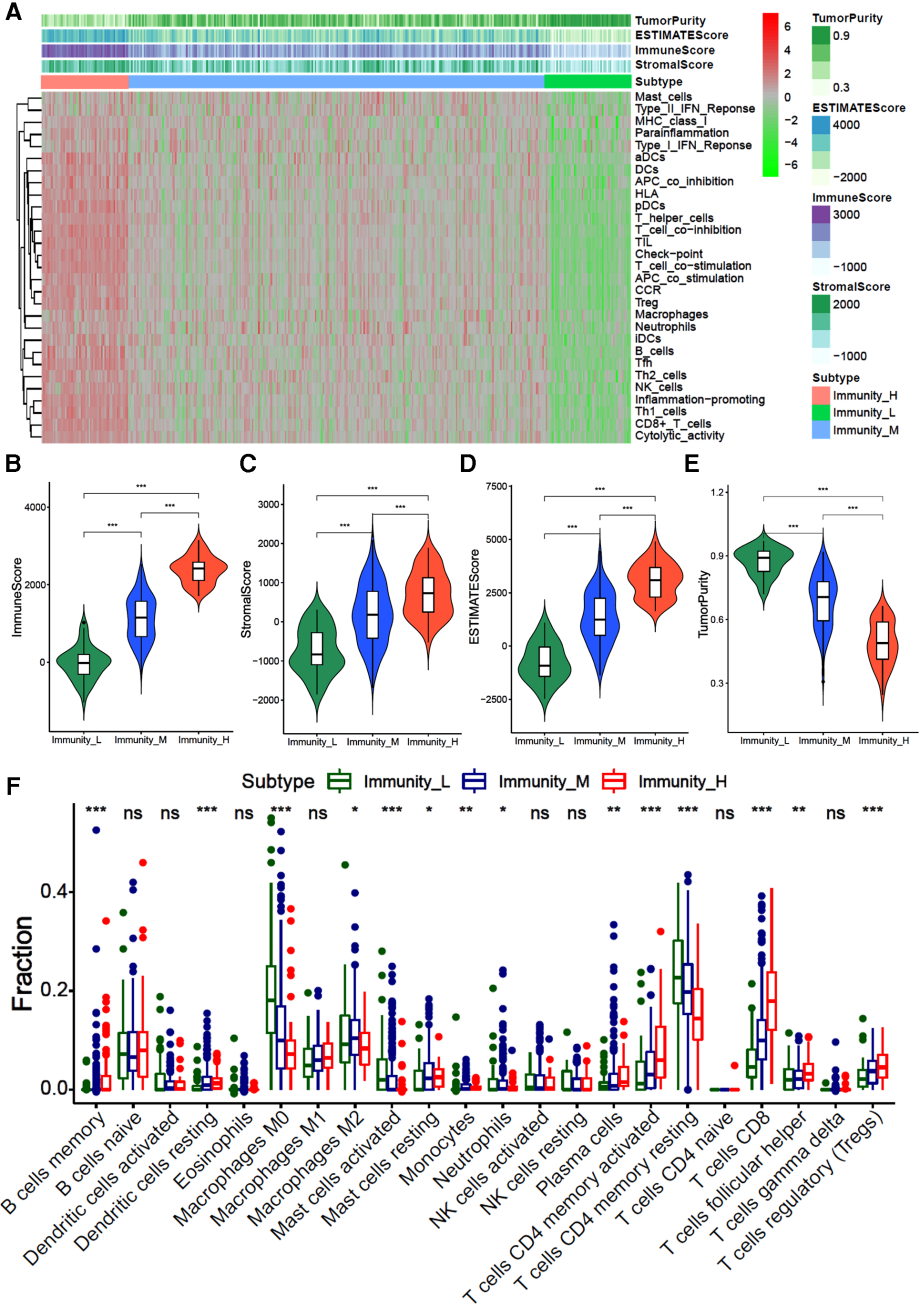
Hierarchical cluster analysis of gastric cancer based on 29 immune-related gene sets (**A**) the heatmap of 29 immune-related gene sets ssGSEA score; (**B–D**). The violin diagram of difference between immunity-H, -M and -L subtypes of gastric cancer in stroma score (**A**), immune score (**B**), total ESTMATE score (**C**) and tumor purity.

We first identified three immune subtypes [immunity-H (*n* = 53, 15.1%), immunity-M (*n* = 248, 70.9%) and immunity-L (*n* = 49, 14.0%)] based on the ssGSEA score of 29 immune-related gene sets by the unsupervised clustering analysis. The heatmap showed that the immunity-H subtype has the highest enrichment score of immune-related genes ssGSEA than the immunity-L and -M subtypes (Figure 1A). The stromal, immune, and total stromal immune score in the immunity-H subtype were significantly higher than the immunity-M and -L subtypes (Figures 1B–D, all *P* < 0.001), while tumor purity showed an opposite trend (Figure 1E, *P* < 0.001). This indicates that the immunity-H GC subtype was associated with the highest infiltration of immune and stromal cells.

We further evaluated the infiltration fraction of 22 immune cells in GC samples by the “CIBERORT algorithm”. The results showed that patients in the immunity-H subtype had the higher fraction of memory B cells, resting dendritic cells, M1 macrophages, resting mast cells, plasma cells, activated CD4^+^ memory T cells, CD8^+^ T cells, and helper T cells than those classified as immunity-L subtype, while lower fraction of M0 macrophages, M2 macrophages and CD4^+^ memory T cells (Figure 1F, all *P* < 0.05). This indicates that the immunity-H GC subtype is dominated by immune positive cells, while the immunity-L subtype is dominated by immune negative cells.

### 3.2. The distribution of clinical characteristics among three immune subtypes

The immunity-H subtype had a relatively large proportion of people over 80 years of age (Supplementary Figure S1A, immunity-H, 8%; immunity-M, 6%; immunity-L, 2%), and higher proportion of gastric corpus tumors (Supplementary Figure S1D, immunity-H, 31%; immunity-L, 24%; immunity-L, 18%), higher proportions of pathological grade 3 (Supplementary Figure S1E, immunity-H, 88%; immunity-M, 56%; immunity-L, 43%). The immunity-H subtype also had higher proportions of diffuse gastric adenocarcinoma (Supplementary Figure S1F, immunity-H, 31%; immunity-M, 16%; immunity-L, 6%), but lower proportion of signet ring cell carcinoma (Supplementary Figure S1F, immunity-H, 13%; immunity-M, 17%; immunity-L, 33%). In addition, patients with advanced TNM stage (stage III/IV) are more likely to present high immunity status (Supplementary Figure S1G, immunity-H, 64%; immunity-M, 54%; immunity-H,41%). Similar results were found in T stage3–4 (Supplementary Figure S1H, immunity-H, 81%; immunity-M, 73%; immunity-H, 67%) and N stage2–3 (Supplementary Figure S1I, immunity-H, 46%; immunity-M, 41%; immunity-H, 30%). There was no significant difference in the distributions of gender and race among the three immune subtypes (Supplementary Figure S1B,C).

### 3.3. Comparison of immune checkpoint and HLA-related genes expression among three immune subtypes

**Figure 2:**
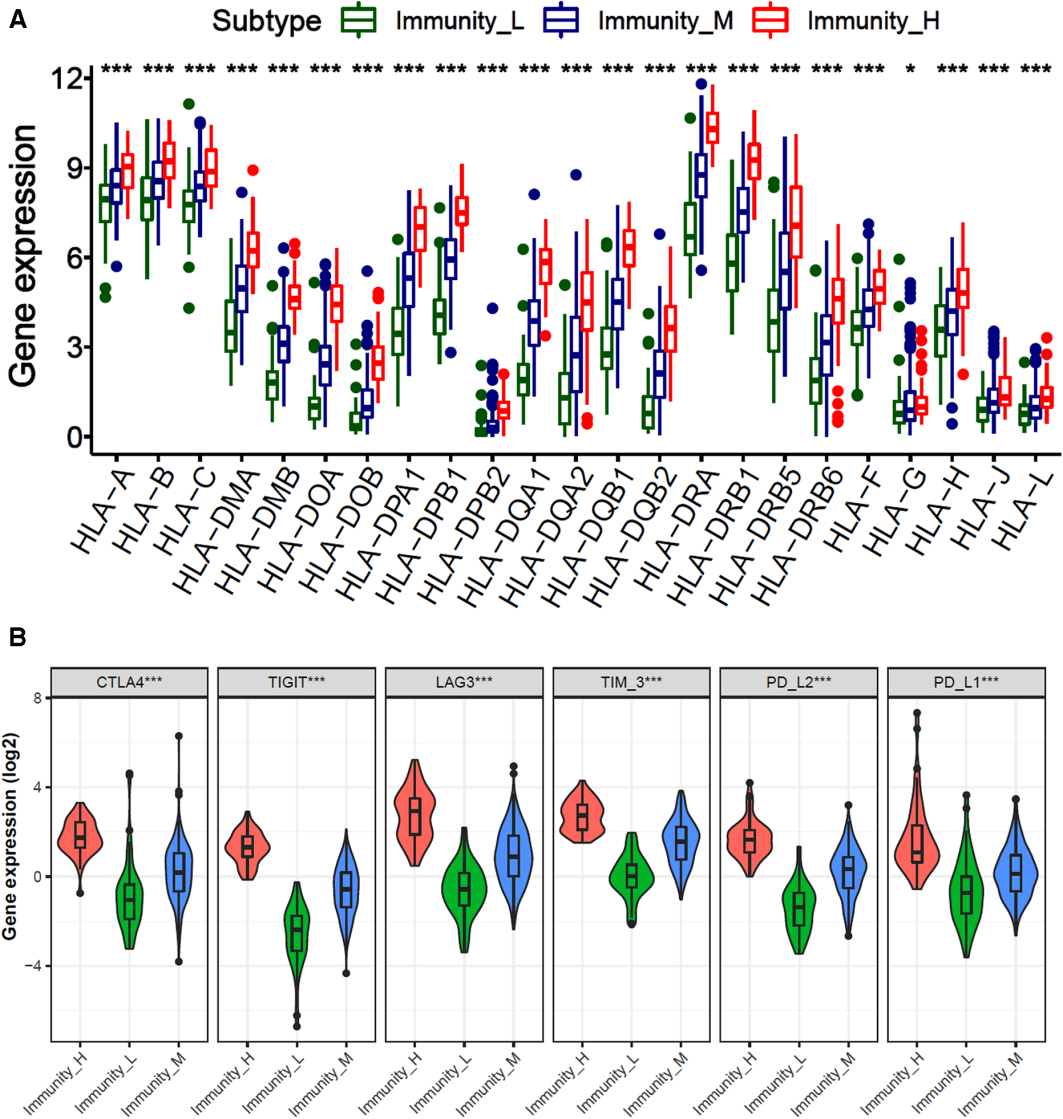
Comparison of HLA and immune checkpoint-related gene expression among three immune subtypes. (**A**) The bar plot of HLA-related genes expression among immunity-H, -M and -L subtypes in gastric cancer. (**B**) Violin diagram of immune checkpoint genes expression in immunity-H, -M and -L subtypes.

To explore the expression of immune-related genes among different immune subtypes of gastric cancer, we analyzed the expression of human leukocyte-associated antigen (HLA) genes and immune checkpoint genes. The results showed that the levels of all HLA genes were the highest expressed in the immunity-H subtype and the lowest in the immunity-L subtype (*P* < 0.001) (Figure 2A). Moreover, the expression of immune checkpoint molecules, such as CTLA4, TIGIT, LAG3, TIM-3, PD-L2, and PD-L1 are also the highest in the immunity-H subtype and the lowest in the immunity-L subtype (Figure 2B). These results indicate that the immune subtypes of GC are significantly correlated with the expression of immune-related genes.

### 3.4. Function enrichment analysis of differentially expressed genes and identification of prognostic-related core genes

**Figure 3:**
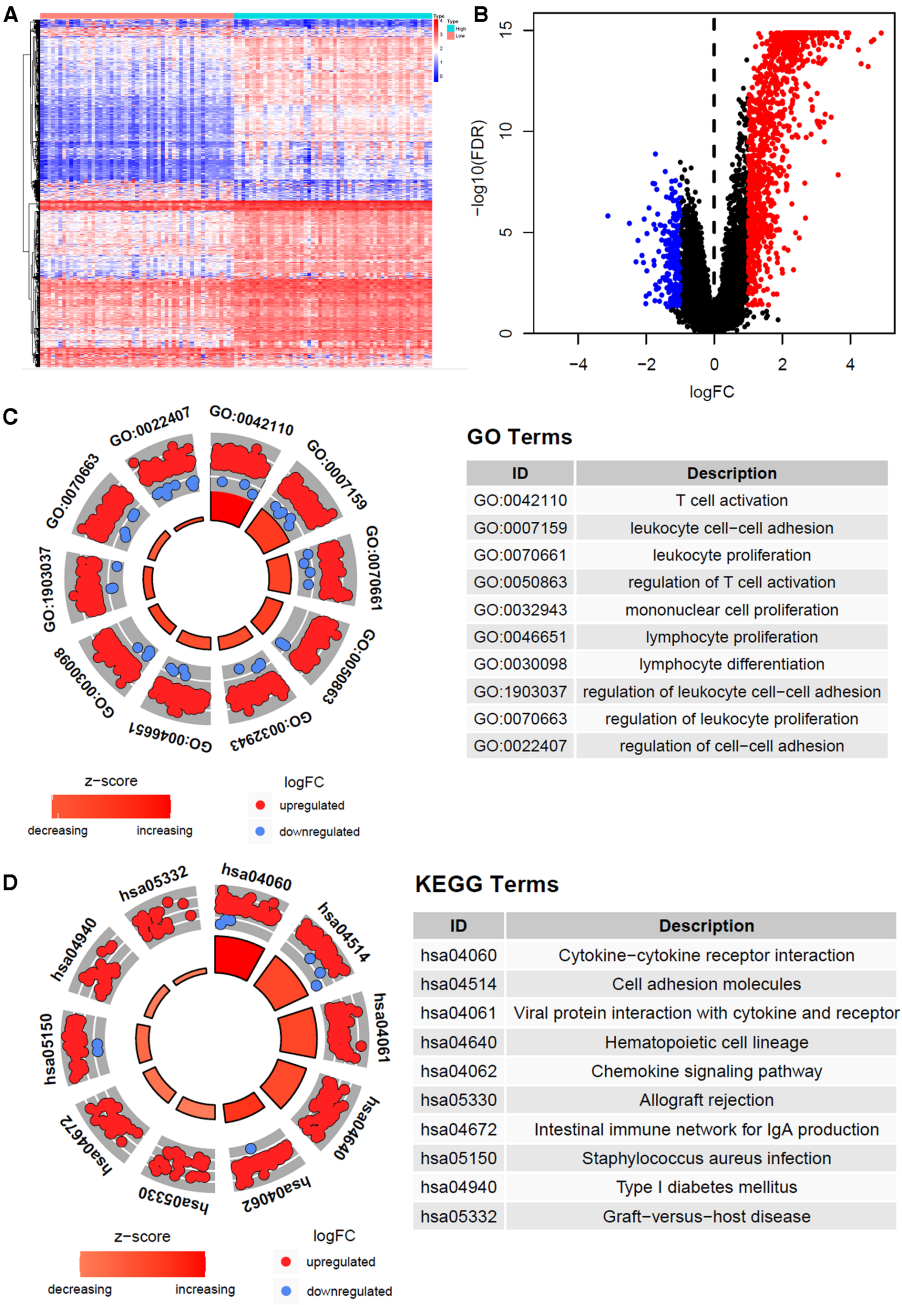
Gene expression heatmap, volcano map and function enrichment analysis of differentially expressed genes. (**A**) The heat map of differentially expressed genes of high and low immune subtypes in gastric cancer (|log FC| > 1, *P* < 0.05). (**B**) Volcano map of differentially expressed genes. (**C**) Differentially expressed genes Circle diagram of GO and KEGG pathway enrichment analysis.

A total of 1226 differentially expressed genes were identified between immune-H and -L subtypes, and are presented with heatmap (Figure 3A) as well as volcano map (Figure 3B). The GO enrichment analysis showed that immune-related pathways such as T cell activation, lymphocyte differentiation, lymphocyte proliferation, cytokine and cytokine receptor interaction and chemokine signaling pathway were significantly enriched in the immunity-H subtype. These results also support the higher immune activity of immunity-H GC subtype.

### 3.5. Construction and validation of prognosis signature based on immune microenvironment for gastric cancer

**Figure 4:**
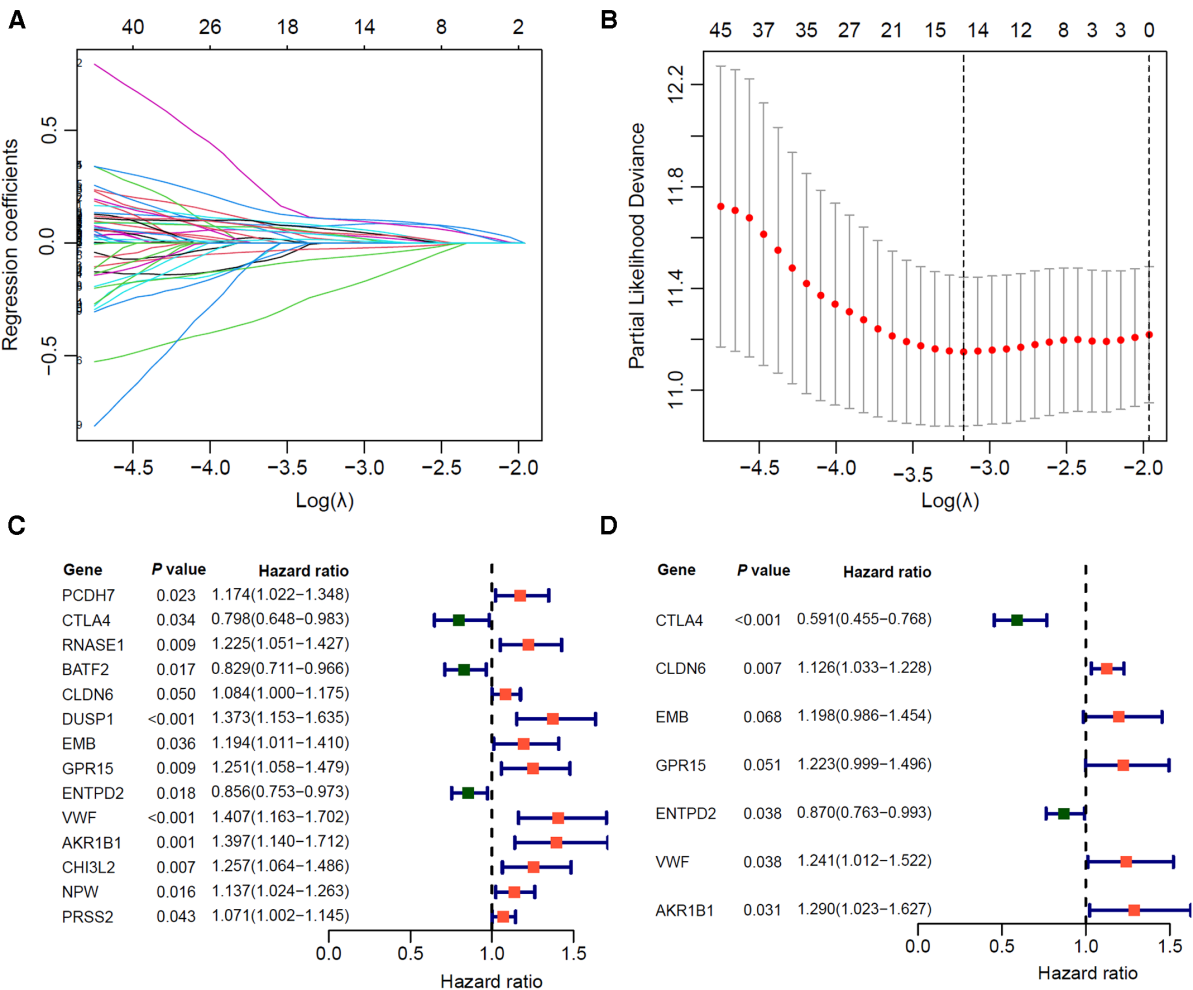
Screening of prognostic related hub genes for gastric cancer in TCGA training cohort. (**A,B**) As the penalty coefficient *λ* increases, the distribution diagram (**A**) and 10-fold cross-validation graph (**B**) of the Lasso-Cox regression coefficient of each variable. (**C**) Forest plot of Univariate COX analysis of 14 lasso genes. (**D**) Multivariate COX analysis forest plot of 7 core prognostic genes.

We randomly divided the 350 GC patients into training (*n* = 246) and testing (*n* = 104) cohorts. The distribution of baseline data was balanced in training and testing cohorts (Table 2). In the TCGA training cohort, we first identified 1,266 differentially expressed genes between immune-H and -L subtypes, and further screened out 90 prognosis-related genes *via* univariate Cox regression (*P* < 0.05). Next, we used lasso-Cox regression to remove the collinearity among genes (Figures 4A,B), and screened out 14 prognostic-related genes, including 3 protective genes and 11 risk genes (Figure 4C). Finally, we identified 7 prognosis-related hub genes by multivariate Cox “stepwise regression” method (Figure 4D).

**Table 2:** Distribution of clinical baseline data of gastric cancer patients in training and testing cohorts.

Variables	Overall	Training cohort	Testing cohort	*P*
	350	246	104	
Age (%)				0.111
<60	106 (30.3)	82 (33.3)	24 (23.1)	
60–69	106 (30.3)	68 (27.6)	38 (36.5)	
70–79	117 (33.4)	79 (32.1)	38 (36.5)	
>80	21 (6.0)	17 (6.9)	4 (3.8)	
Gender (%)				0.873
Male	226 (64.6)	160 (65.0)	66 (63.5)	
Female	124 (35.4)	86 (35.0)	38 (36.5)	
Origin (%)				0.218
Body	84 (24.0)	56 (22.8)	28 (26.9)	
Cardia	86 (24.6)	66 (26.8)	20 (19.2)	
Fundus	40 (11.4)	23 (9.3)	17 (16.3)	
Gastric antrum	127 (36.3)	91 (37.0)	36 (34.6)	
Other	13 (3.7)	10 (4.1)	3 (2.9)	
Grade (%)				0.426
G1	9 (2.6)	7 (2.8)	2 (1.9)	
G2	125 (35.7)	83 (33.7)	42 (40.4)	
G3	207 (59.1)	148 (60.2)	59 (56.7)	
GX	9 (2.6)	8 (3.3)	1 (1.0)	
TNM_staage (%)				0.202
NA	14 (4.0)	10 (4.1)	4 (3.8)	
stage I	46 (13.1)	29 (11.8)	17 (16.3)	
stage II	110 (31.4)	76 (30.9)	34 (32.7)	
stage III	143 (40.9)	99 (40.2)	44 (42.3)	
stage IV	37 (10.6)	32 (13.0)	5 (4.8)	
T_stage (%)				0.369
T1	16 (4.6)	9 (3.7)	7 (6.7)	
T2	74 (21.1)	48 (19.5)	26 (25.0)	
T3	161 (46.0)	118 (48.0)	43 (41.3)	
T4	95 (27.1)	69 (28.0)	26 (25.0)	
TX	4 (1.1)	2 (0.8)	2 (1.9)	
N_stage (%)				0.139
N0	103 (29.4)	64 (26.0)	39 (37.5)	
N1	93 (26.6)	73 (29.7)	20 (19.2)	
N2	72 (20.6)	53 (21.5)	19 (18.3)	
N3	71 (20.3)	49 (19.9)	22 (21.2)	
NX	11 (3.1)	7 (2.8)	4 (3.8)	
M_stage (%)				0.138
M0	312 (89.1)	214 (87.0)	98 (94.2)	
M1	25 (7.1)	21 (8.5)	4 (3.8)	
MX	13 (3.7)	11 (4.5)	2 (2.0)	

NA, not available.

**Figure 5:**
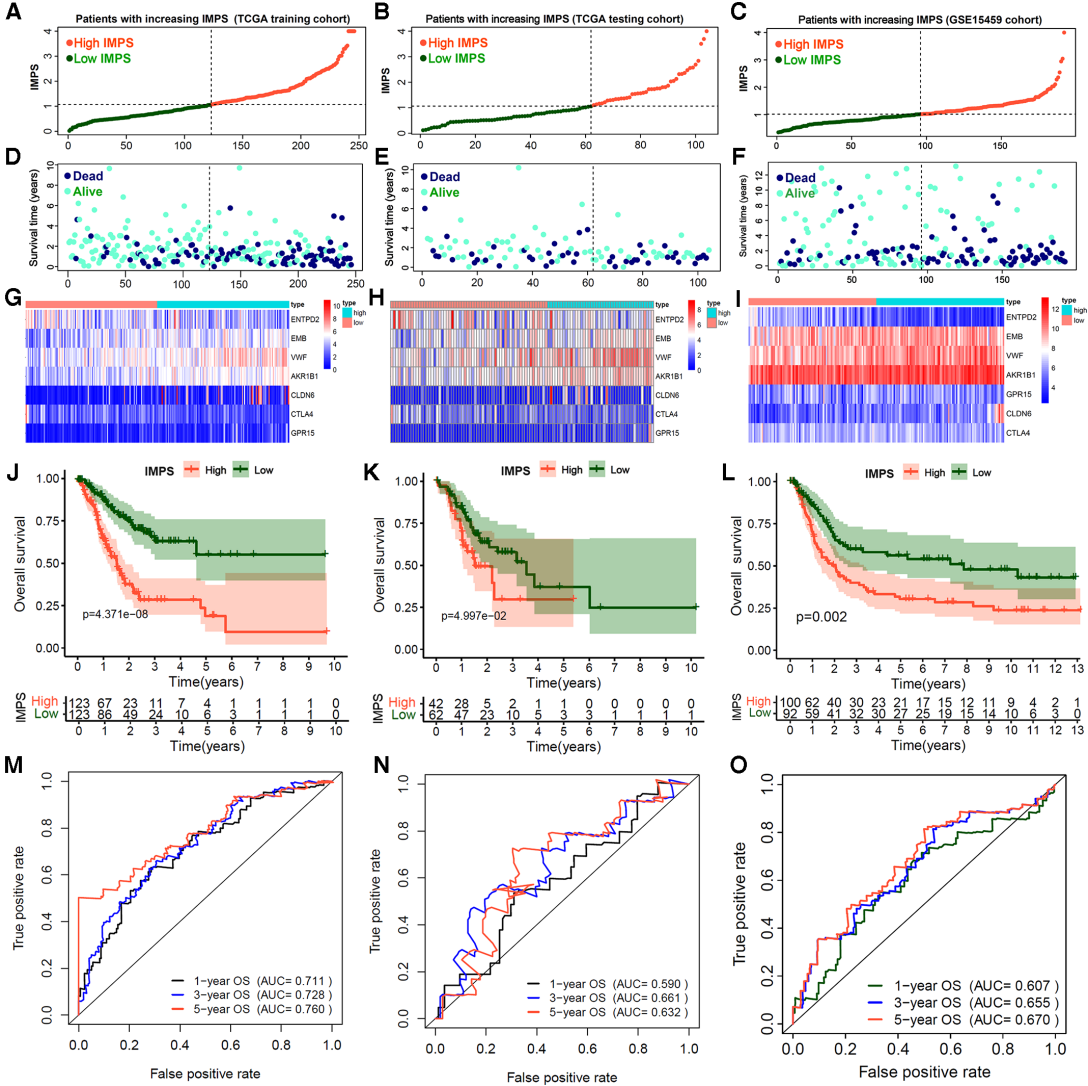
Construction and validation of immune microenvironment-related prognosis signature (IMPS) for gastric cancer gastric. (**A–F**) Distribution scatter plots of patient survival status increasing with IMPS in TCGA training (**A,D**), TCGA testing (**B,E**) and GSE15459 (**C,F**) independent validation cohorts. (**G–I**). The expression heatmap of 7 hub genes in TCGA training (**G**), TCGA testing (**H**) and GSE15459 (**I**) cohorts. (**J–L**) Kaplan-Meier analysis of patients between high- and low-IMPS in TCGA training (**J**), TCGA testing (**K**) and GSE15459 (**L**) cohorts. (**M–O**). Time-ROC curves of 1-, 3- and 5-year overall survival in TCGA training (**M**), TCGA testing (**N**) and GSE15459 (**O**) cohorts.

Based on the expression level and regression coefficients of the seven core genes, the formula of IMPS was shown in the Methods section. Using the median IMPS of 1.07 as the cut-off value for the prognostic risk of gastric cancer, the patients were divided into the high- and low-IMPS groups (Figure 5A). Next, we observed that the proportion of death gradually increased and the survival time gradually decreased as IMPS score increased (Figure 5D), which indicated that the IMPS was closely related to the prognostic risk of GC in the real world. We further explored the association between IMPS and the 7 prognostic hub gene expression. The expression heatmap showed that the expression of VWF and AKR1B1 was positively correlated with IMPS (Figure 5G). Similar phenomena were also observed in the test set and the GEO independent validation set (testing cohort: Figures 5B,E,H; GSE15459 cohort: Figures 5C,F,I). In addition, the GC patients in the high-IMPS group had poorer survival in the training cohort than the low-IMPS group (Figure 5J; *P* < 0.001), with consistent conclusions in the testing cohort (Figures 5K,L; both with *P* < 0.01). To reduce bias, we further validated our model in GSE62254 (Supplementary Figure S2A), GSE57330 (Supplementary Figure S2B) and GSE34942 (Supplementary Figure S2C) independent cohorts. We explored the predictive value of IMPS in the outcome of immunotherapy response in IMvigor210 and Riaz-2017 cohorts. The patients in low-IMPS subgroup have a better OS rate and higher response fraction than the high-IMPS subgroup (Supplementary Figures S2C–F).

Finally, we evaluated the accuracy of the IMPS in predicting 1-year, 3-year and 5-year OS of GC by the survival ROC curve. The prediction accuracy AUCs in the training cohort were 0.711, 0.728 and 0.760, respectively; in the testing cohort 0.590, 0.661 and 0.632, respectively; and in the GSE15459 cohort 0.607, 0.655 and 0.670, respectively. Furthermore, the multivariate Cox analysis showed that the IMPS was an independent risk factor for GC prognostic after adjustment by age, gender, grade and TNM stage (Table 3, *P* < 0.001). These findings suggest that the IMPS we identified has moderate predictive value in GC prognostic risk assessment.

**Table 3:** Multivariate Cox analysis of IMPS and clinical features for gastric cancer prognosis.

Variables	HR	HR (95% CI)	*P*-value
**IMPS** (Low)	Ref.		
High	2.88	4.14–2.00	<0.001
**Age** (<60)	Ref.		
60–69	1.65	1.03–2.62	0.037
70–79	2.25	1.45–3.50	<0.001
>80	2.34	0.96–5.69	0.061
**Gender** (Female)	Ref.		
Male	1.33	0.92–1.91	0.128
**Grade** (G1)	Ref.		
G2	2.96	0.40–21.73	0.286
G3	4.01	0.56–29.85	0.164
**TNM** (Stage I)	Ref.		
Stage II	1.26	0.64–2.46	0.502
Stage III	1.94	1.03–3.64	0.039
Stage IV	3.09	1.49–6.39	0.003

HR, hazard rate; CI, confidence interval.

**Figure 6:**
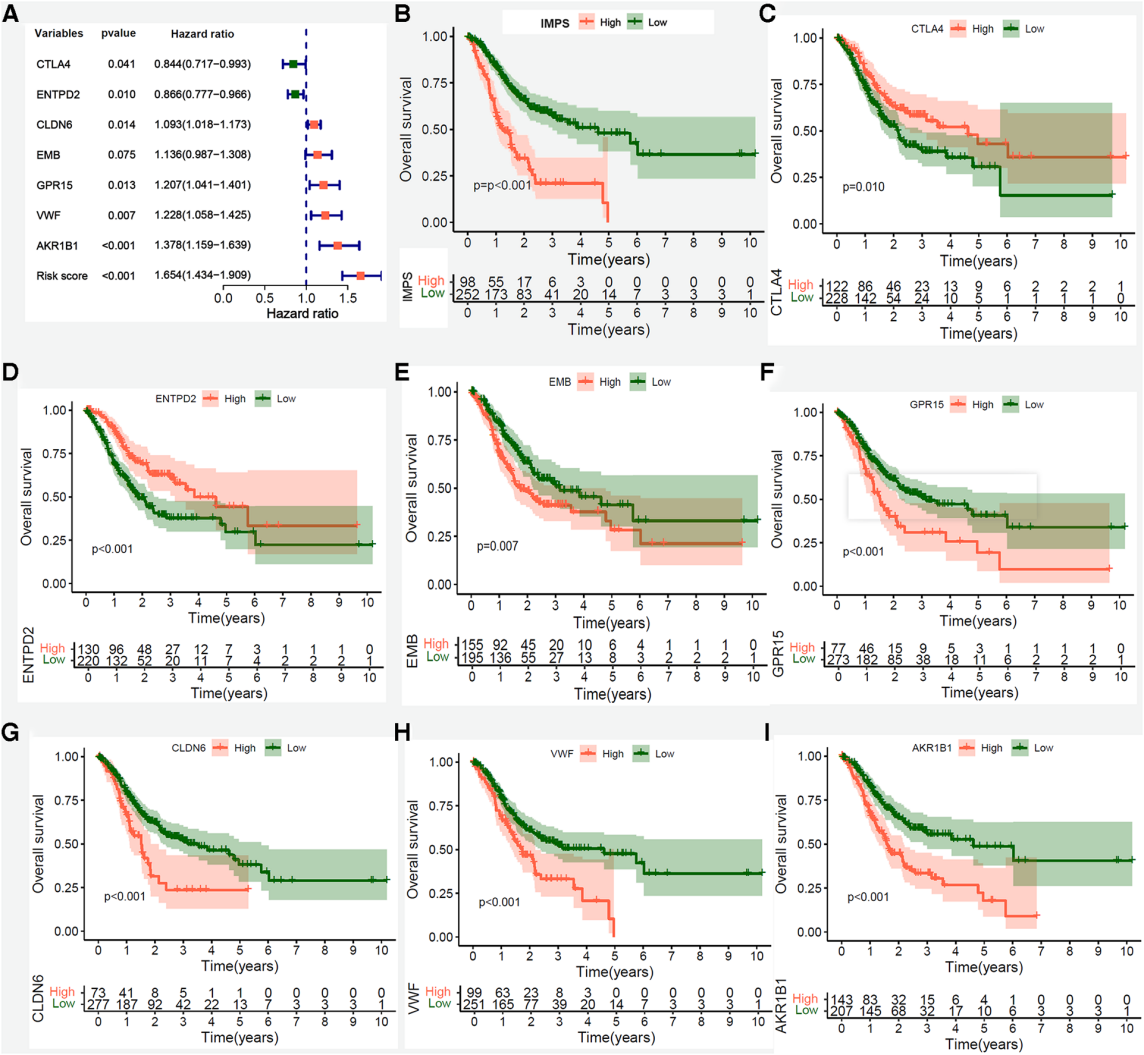
Kaplan-Meier survival analysis of IMPS and 7 prognostic hub genes in the entire TCGA-STAD cohort. (**A**) Univariate Cox forest-plot of IMPS and 7 prognostic hub genes. (**B–G**) Kaplan–Meier curve survival analysis of IMPS and 7 core prognostic gene, red line means high expression group, blue line means low expression group. (**B**) IMPS, (**C**) CTLA4, (**D**) ENTPD2, (**E**) EMB, (**F**)GPR15, (**G**) CLDN6, (**H**) VWF and (**I**) AKR1B1.

To further explore the prognosis value of 7 hub genes, we performed Kaplan-Meier curve survival analysis in the TCGA-STAD, GSE15459 and GSE57303 cohorts. The results showed that patients in the TCGA-STAD cohort with high expression of CTLA4 and ENTPD2 had a better OS (Figures 6A–C, both *P *< 0.05), whereas patients with high expression of EMB, GPR15, CLDN6, VWF and AKR1B1 had a poorer OS (Figures 6D–I, all *P *< 0.05). These findings were further confirmed in GSE15459 and GSE57303 cohorts (Supplementary Figures S3A–N). In addition, we performed RT-PCR to explore the differential expressions between human GC (MKN45 and AGS) and normal gastric mucosal epithelial cell lines (GES-1). The RT-PCR results showed that the CTLA4 and ENTPD2 were highly expressed in normal gastric mucosal epithelial cell lines (GES-1), while the EMB, CLDN6, VWF and AKR1B1 were highly expressed in human GC cell lines (MKN45 and AGS), and the expression of GPR15 had no significant difference between normal and tumor cell lines (Supplementary Figure S4A). We further preformed the PCR verification of seven Hub genes in five paired benign and tumor gastric tissue samples. Compared with benign tissues, the CTLA4 and ENTPD2 were highly expressed in gastric tumor tissues, while the AKR1B1, CLDN6, EMB, GPR15 and VWF were highly expressed (Supplementary Figures S4B,C).

### 3.7. Correlation analysis of IMPS with clinical characteristics and somatic mutations

**Figure 7:**
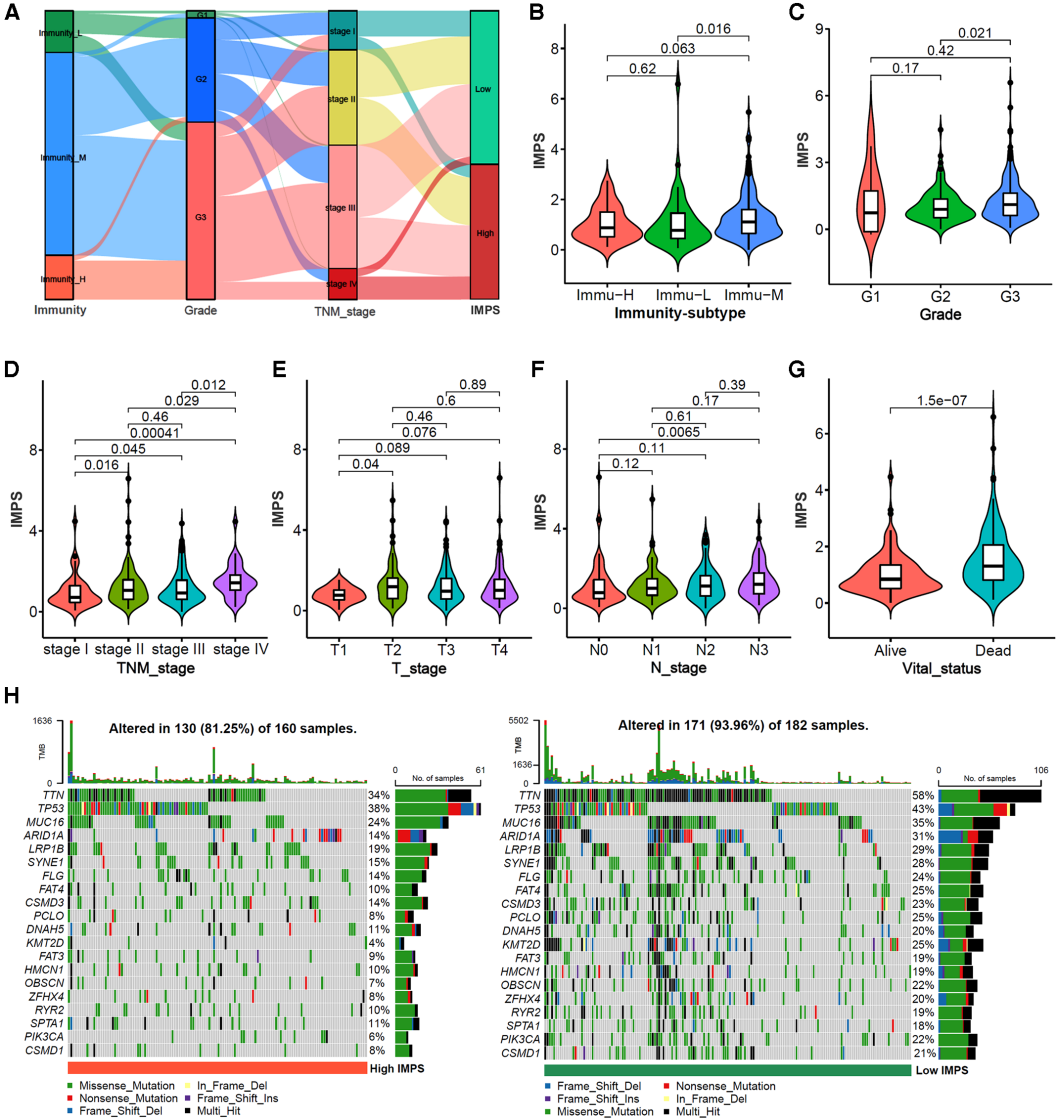
Correlation analysis of IMPS with clinical characteristics and somatic mutations. (**A**) Alluvial diagram of immunity-subtypes with different pathological grades, TNM stages and IMPS subgroups. (**B–G**) Comparison of IMPS among different immune subtypes, pathological grades, T stages, N stages and vital status. (**H**) mutation landscape of gastric cancer between high-IMPS (left) and low-IMPS (right) subtypes.

The alluvial diagram of the immunity subtypes with different pathological grades, TNM stages and IMPS subgroups (Figure 7A) indicated that the immunity-H subtype was more likely linked to the higher grade, more advanced TNM stages and higher IMPS; while the immunity-L subtype exhibited a lower grade, earlier TNM stages and lower IMPS. The GC patients in immunity-M subtype showed higher IMPS than those in immunity-L and immunity-H subtypes (Figure 7B, *P *= 0.016 and *P *= 0.063). GC patients with higher IMPS were also more likely to be associated with higher grade, more advanced TNM stages, higher T and N stage, and death (Figures 7C–F, all *P* < 0.001). The mutation landscape showed that the patients in the IMPS-H subtype had a higher mutation alter ratio (81.25%) than the IMPS-L subtype (93.96%) with higher tumor mutation burden (Figure 7H).

### 3.8. Construction and evaluation of the combined nomogram considering IMPS and clinical characteristics

**Figure 8:**
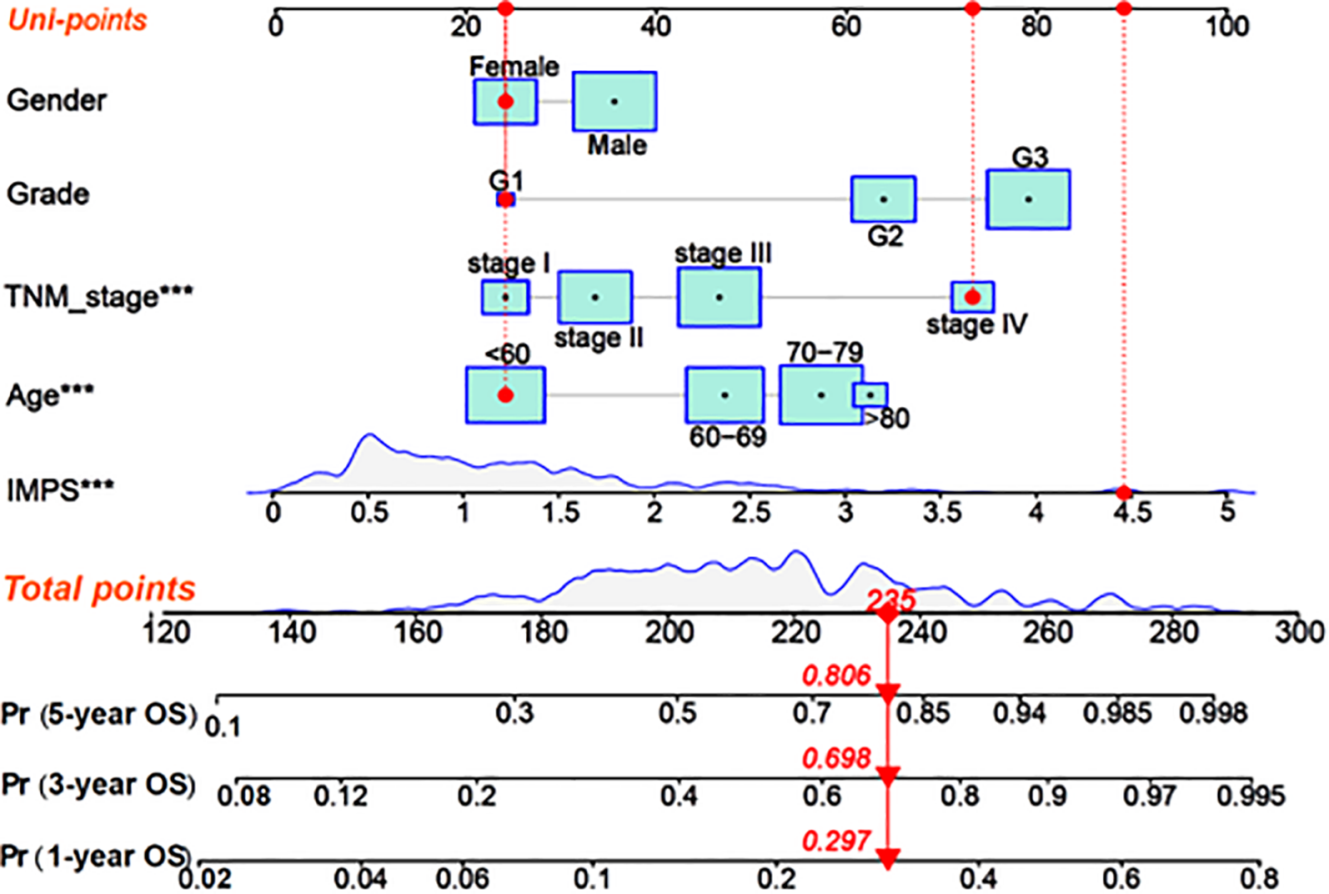
Construction of the combined nomogram based on IMPS and clinical characteristics. Nomogram combined IMPS and clinical characteristics for predicting 1-year, 2-year, and 3-year OS. The red dot presented a sample with each variable of the combined nomogram. The total point of the nomogram was calculated as the sum of each univariable risk points (Uni-points).

**Figure 9:**
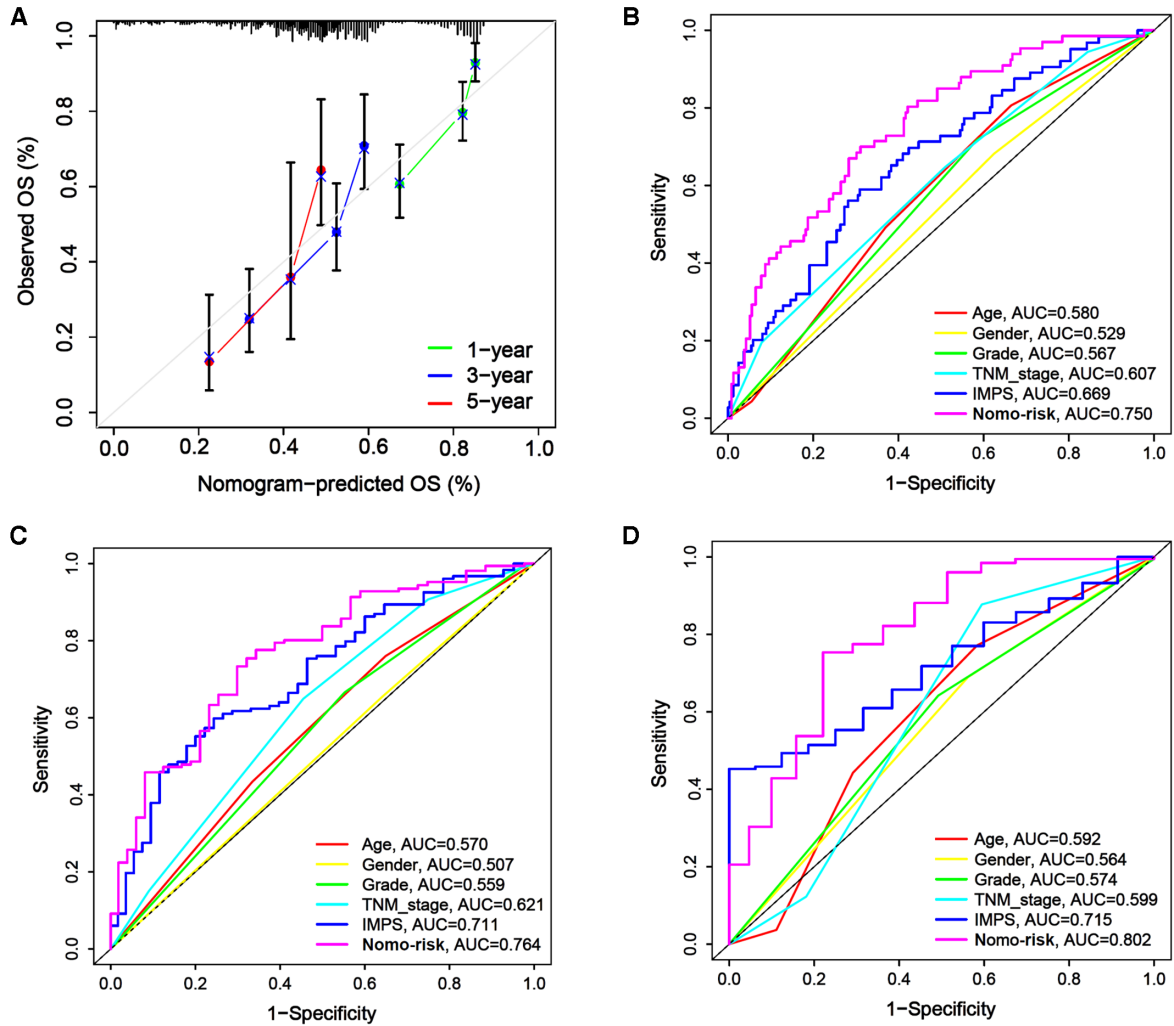
Calibration curves and time-ROC curve of the combined nomogram. (**A**) The combined nomogram calibration curves for 1-year, 2-year, and 3-year consistency between the predicted and observed OS. The actual performance of our combined nomogram is shown as the blue line. (**B–D**) The time-ROC curves of the combined nomogram risk, IMPS, TNM stage, grade, age, and gender in predicting OS at 1 (**B**), 3 (**C**), and 5 (**D**) years.

In order to enhance the prediction performance, we further constructed a combined nomogram considering IMPS and clinical characteristics (Figure 8). The nomogram showed that patient with higher TNM stage, older age and higher IMPS was significantly correlated with poorer prognosis. We further performed the calibration curve and time-ROC curve to present the consistency and prediction value of the combined nomogram with the actual observed OS of 1, 3, and 5 years. The calibration curve exhibited high consistency of OS prediction with the actual observation (Figure 9A).

The combined nomogram (AUC = 0.750) showed the highest value of the 1-year OS prediction (Figure 9B), followed by IMPS (AUC = 0.669), TNM stage (AUC = 0.607), age (AUC = 0.580), grade (AUC = 0.567) and gender (AUC = 0.529). The AUC values of the combined nomogram in predicting 3-year (AUC = 0.764) and 5-year (AUC = 0.802) OS were also higher than those with IMPS and individual clinical characteristics (Figures 9C,D). These results indicate that the combined nomogram model is more accurate than IMPS and the individual clinical characteristics of TNM stage, age, grade and gender in predicting the OS of GC patients.

## 4. Discussion

In recent years, the GC classification based on multi-omics analysis has been extensively studied, and these efforts may lay the foundation for the development of novel GC biomarkers and drug targets (24–26). It has also been demonstrated that the TME components play a crucial role in tumor development. However, the tumor-immune interactions have not been fully understood. In the present study, we identified three immunophenotypes (immunity-H, -M and -L) based on the characteristics of 29 immune-related gene sets. In addition, we constructed an immune microenvironment-related prognosis signature (IMPS) *via* univariate Cox regression, Lasso-Cox regression, and multivariate Cox regression, and validated in the testing and independent cohorts. The patients with higher IMPS were associated with higher grade, advanced TNM stages, higher T and N stage, and death. We further constructed a nomogram model combining IMPS and clinical variables. The IMPS and the combined nomogram model have potential value in predicting survival outcomes of gastric cancer.

We further constructed a 7-gene (CTLA4, CLDN6, EMB, GPR15, ENTPD2, VWF and AKR1B1) prognosis signature, IMPS, based on the immune microenvironment and screened by univariate Cox regression, Lasso-Cox regression, and multivariate Cox regression. In this study, the gastric cancer patients with high expression of CTLA4 and ENTPD2 had a better survival prognosis, with high expression of CLDN6, EMB, GPR15, VWF and AKR1B1 suggesting poor prognosis, which was consistent with previous studies. CTLA4 is an immune checkpoint gene, and drugs of CTLA4 inhibitors have been used in clinical practice (27, 28). In addition, CTLA4 is mainly expressed in the T cells’ cytoplasm, and its membrane levels are changing dynamically during T cell activation (29). By inhibiting the expression of CTLA4 in tumors, it can increase the antigen presentation of CD4^+^ T cells, and improve the killing effect of CD8^+^ T cells on tumors. Patients with high CTLA4 expression potentially benefit from treatment with CTLA4 inhibitors (30). Overexpression of nucleoside triphosphate diphosphate hydrolase 2 (ENTPD2) is an indicator of poor prognosis in HCC, Chiu DK et al. found that in HCC, ENTPD2 converts extracellular ATP into 5′-AMP, prevents myeloid-derived suppressor cells (MDSC) differentiation, promotes MDSC maintenance (31), and allows HCC cells to escape immune surveillance (32), but it has not been reported in gastric cancer. In this study, we found that elevated ENTPD2 expression suggests better overall survival, which may be related to cancer type differences and internal heterogeneity of tumors.

Claudin6 (CLDN6) is a member of the tight junction family and is involved in intercellular adhesion (33). Yu S et al. found that CLDN6 can affect EMT process by affecting YAP1 and YAP1-snail1 axis, and promote gastric cancer proliferation and invasion (34). Embigin (EMB), a transmembrane glycoprotein of the immunoglobulin superfamily (35), is involved in the occurrence and development of prostate cancer, pancreatic cancer and breast cancer, and is associated with poor prognosis in cancer patients (36–38), but not in reports of gastric cancer. G protein-coupled receptor 15 (GPR15) is an unconventional chemokine receptor that mediates T_reg_ homing and immunosuppression (39) by directing T_reg_ into the colon, thereby altering the tumor microenvironment and promoting occurrence of intestinal tumors (40). Von Willebrand factor (VWF) is a potent regulator of angiogenesis, tumor growth, and metastasis, and gastric cancer-related plasma VWF activity levels are significantly elevated in advanced disease stages (41). Aldehyde-ketoreductase family 1 member B1 (AKR1B1) is overexpressed in a variety of tumors and is involved in inflammation, cell cycle, epithelial-to-mesenchymal transition, cell survival, and apoptosis (42). In GC, the expression of AKR1B1 is significantly correlated with the clinicopathological characteristics, and the patients with low AKR1B1 have a better OS than that in patients with high AKR1B1 (43).

We constructed an immune microenvironment-related prognosis signature (IMPS) in the training cohort, and further validated it in the testing and independent cohorts. The GC patients with higher IMPS were associated with higher TNM stages and had a bad prognosis. And the reliability of IMPS was validated in the TCGA testing and three independent GEO cohorts. We further a nomogram combined with age, gender, pathology grade and TNM stage. The combined nomogram showed best performance in OS time prediction outperforms IMPS and the individual clinical parameters. The IMPS and the combined nomogram model have potential value in predicting survival outcomes of gastric cancer. Although we collected multiple datasets and conducted comprehensive analyses, this study still needs to be validated by prospective clinical studies with large samples.

## 5. Conclusions

In summary, our study constructed a prognostic signature (IMPS) based on the immune microenvironment, and further constructed a combined nomogram based on IMPS and clinical characteristics. The IMPS and the combined nomogram were well-performed in predicting the 1-, 2- and 5-year overall survival prognosis, and might provide important value for the diagnosis and treatment of gastric cancer.

## Data Availability

The original contributions presented in the study are included in the article/**Supplementary Material**, further inquiries can be directed to the corresponding author.
