# Supplementary material for: Construction and validation of a prognosis signature based on the immune microenvironment in gastric cancer
Source: Front Surg. 2023 Mar 31;10:1088292. doi: 10.3389/fsurg.2023.1088292 (PMC10102374; doi:10.3389/fsurg.2023.1088292)
Supplement: Supplementary file 2 [file Presentation1.pdf]

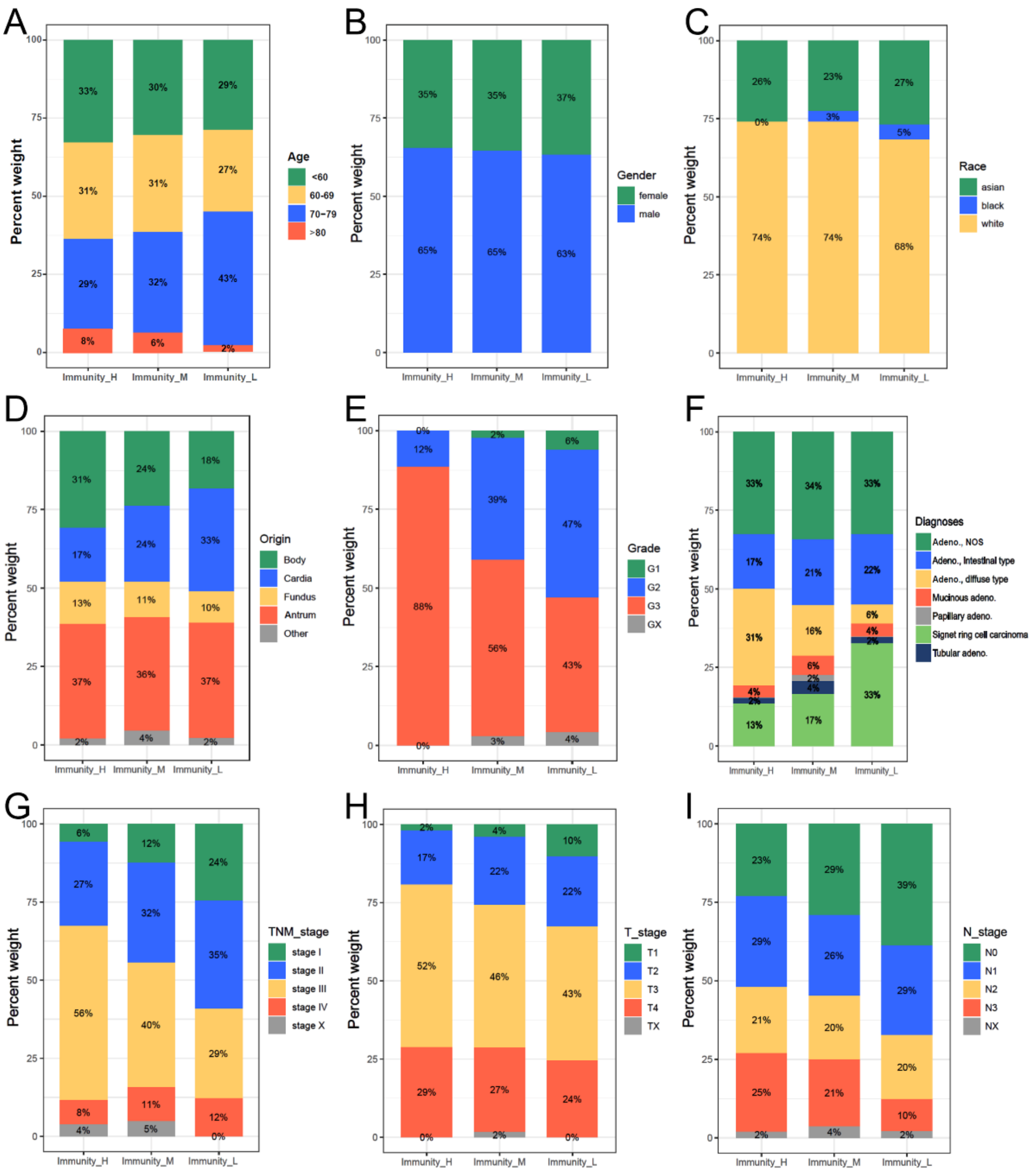

Figure S1. Distribution of clinical features among three immune subtypes

(A-I) The precent weight of age (A), gender (B), race (C), origin site (D), grade (E), pathological diagnosis (F), TNM stage (G), T stage (H) and N stage among three immune subtypes (immunity-H, -M and -L).

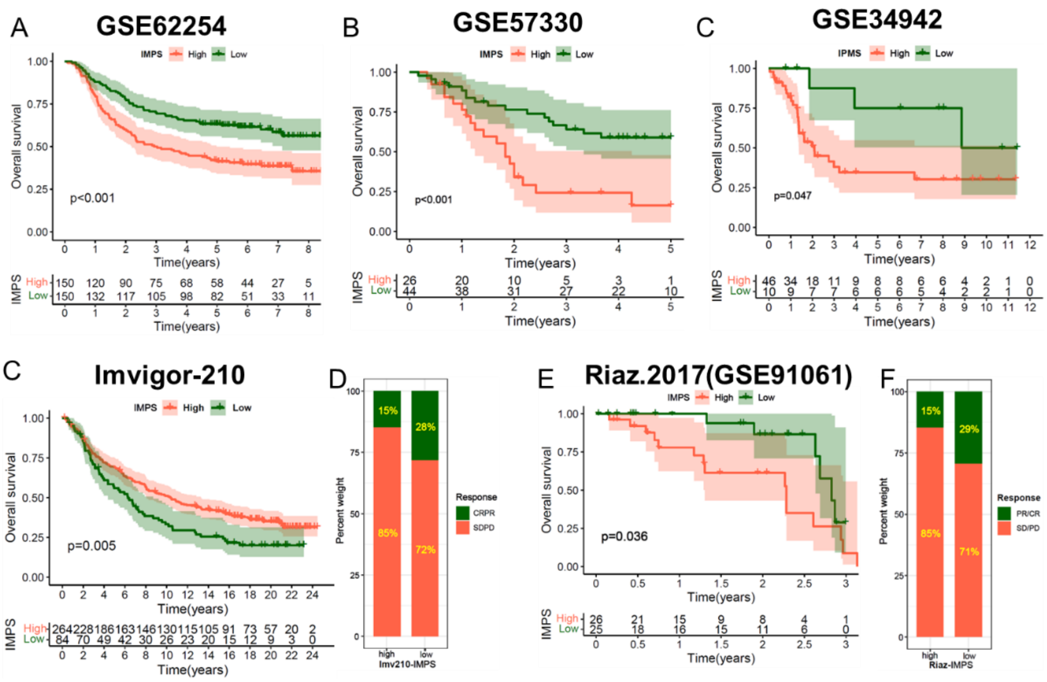

Figure S2. Overall survival analysis of IMPS in GC and immunotherapy datasets (A-B) Kaplan-Meier analysis of IMPS in gastric cancer (GSE15459 and GSE57303) cohorts. (C-D) Kaplan-Meier analysis of IMPS in immunotherapy cohorts, IMvigor210 cohort (metastatic urothelial carcinoma) and Riaz-2017 cohort (advanced melanoma).

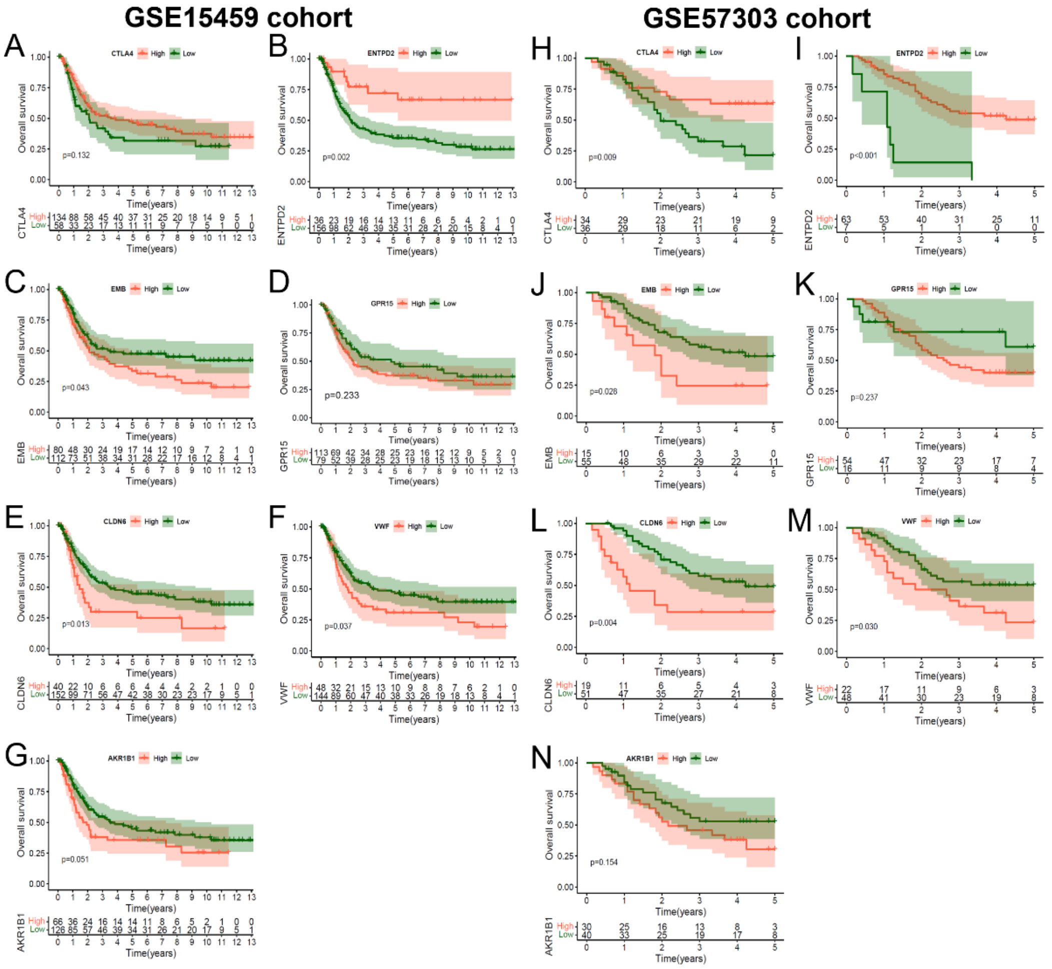

Figure S3. Overall survival analysis of 7 hub genes in GSE15459 and GSE57303 cohorts
(A-G) Kaplan-Meier analysis of CTLA-4 (A), ENTPD2 (B), EMB(C), GRP15(D), CLDN6(E), VWF(F) and (G) in GSE15459 cohort. (H-N) Kaplan-Meier analysis of CTLA-4 (H), ENTPD2 (I), EMB(J), GRP15(K), CLDN6(L), VWF(M) and (G) in GSE15459 cohort.

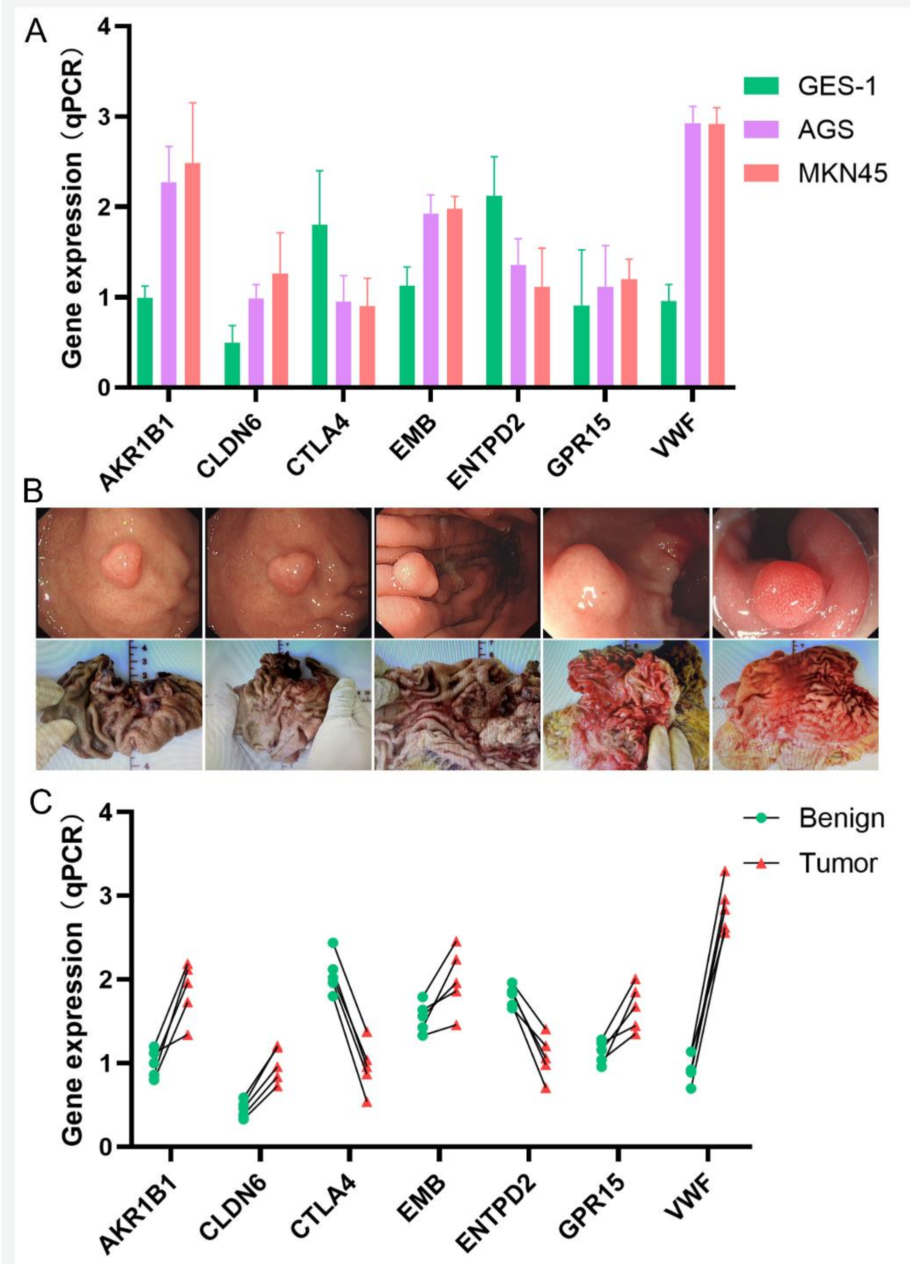

Figure S4. Expression validation of 7 hub genes in cell lines and tissues

(A) Expression comparison of 7 hub genes between gastric cancer and normal gastric epithelial cell lines. The normal gastric epithelial cell line, GES-1, was presented in green bar, and the two gastric cancer cell lines were presented in purple bar (AGS) and red bar (MKN45), respectively. (B) The gross image of five paired benign and GC tissue samples used for qPCR verification. (C) Expression comparison of 7 hub genes between paired benign and GC tissue samples. The benign tissue was presented in green dots, and the gastric cancer tissue was presented in red dots, respectively.
